# Inhibition of Human Coronavirus 229E by Lactoferrin-Derived Peptidomimetics

**DOI:** 10.3390/pharmaceutics17081006

**Published:** 2025-08-01

**Authors:** Maria Carmina Scala, Magda Marchetti, Martina Landi, Marialuigia Fantacuzzi, Fabiana Superti, Mariangela Agamennone, Pietro Campiglia, Marina Sala

**Affiliations:** 1 Department of Pharmacy, University of Salerno, Via Giovanni Paolo II 132, 84084 Fisciano, Italy; mscala@unisa.it (M.C.S.); martlandi@unisa.it (M.L.); pcampiglia@unisa.it (P.C.); 2 National Centre for Innovative Technologies in Public Health, National Institute of Health, Viale Regina Elena 299, 00161 Rome, Italy; magda.marchetti@iss.it; 3 Department of Pharmacy, “G. d’Annunzio” University of Chieti-Pescara, Via dei Vestini 31, 66100 Chieti, Italy; marialuigia.fantacuzzi@unich.it (M.F.); mariangela.agamennone@unich.it (M.A.); 4 Association for Research on Integrative Oncology Therapies (ARTOI) Foundation, Via Ludovico Micara 73, 00165 Rome, Italy

**Keywords:** lactoferrin, peptide, coronaviruses, spike, docking, peptidomimetics

## Abstract

**Background/Objectives:** Viral respiratory infections have a significant impact on global health and the economy. While vaccines are effective in preventing infection, they might not be available or sufficient when used alone and must be complemented by specific therapeutic strategies. The development of new antiviral agents is increasingly important due to the continual emergence of novel respiratory pathogens. Previously we identified bovine lactoferrin (bLf)-derived tetrapeptides and peptidomimetics that showed potent in vitro activity against the influenza A virus in the picomolar range. **Methods:** Inspired by these results, in this study, we evaluated the antiviral potential of these compounds against HCoV-229E, a human coronavirus that can cause severe disease in immunocompromised individuals, using a compound repositioning approach. **Results:** Functional studies revealed that SK(N-Me)HS (**3**) interferes with viral entry and replication, while compound S*N*KHS (**5**) primarily blocks infection in the early stages. Biophysical analyses confirmed the occurrence of high-affinity binding to the viral spike protein, and computational studies suggested that the compounds target a region involved in conformational changes necessary for membrane fusion. **Conclusions:** These findings highlight these compounds as promising candidates for coronavirus entry inhibition and underscore the value of compound repurposing in antiviral development.

## 1. Introduction

Respiratory infections are a major cause of morbidity and mortality worldwide [1]. The viral pathogens representing the main causes of upper and lower respiratory tract illnesses in the winter season are severe acute respiratory syndrome coronavirus 2 (SARS-CoV-2), influenza virus (IAV), human respiratory syncytial virus (RSV), and other viruses mainly responsible for upper respiratory infections (URIs). For SARS-CoV-2, IAV, and RSV, vaccines are the first line of defense, while there are no vaccines for the other ones.

All known respiratory viruses can cause URIs, also called the “common cold” [2]. These infections represent the most common acute human disease worldwide [3] and are responsible for a significant economic burden on society in terms of the cost of medications, visits to health care providers, and absenteeism.

URIs frequently involve co-infections with multiple viruses or a combination of viral and bacterial pathogens, which can influence the disease severity and clinical outcomes [4].

The relative proportions of different types of viruses that cause URIs may vary depending on the season and the methods of viral sampling and detection. Common cold coronaviruses cause about 30 percent of upper respiratory infections, although most of the numerous epidemiological studies report considerable variability in the rate of infections by different HCoVs [5,6,7]. The most common strains are HCoV-OC43 and HCoV-229E. Both have been reported to show marked seasonality, with infections being more common during the colder months. Both cause mild-to-severe respiratory infections, including pneumonia and bronchiolitis [8], and are the second most frequently detected agents causing the common cold, after Rhinovirus [9,10]. The predictable seasonality of respiratory viruses depends strongly on the geographic location and climate. In particular, in temperate regions of the Northern and Southern Hemispheres, their annual seasonal pattern is restricted to a few months during the winter [11]. As a matter of fact, in a study on respiratory infections caused by HCoV conducted in Argentina, HCoV-OC43 and HCoV-229E were detected during the cold months (March to August) as being responsible for single or multiple infections. Although infections occurred primarily in children, HCoV-229E was also detected in adults and was significantly associated with asthma [10]. Additionally, HCoV-229E has been associated with pathologies such as pneumonia in immunocompromised individuals [12]. HCoV-229E, sharing a close evolutionary history and important physicochemical characteristics with highly pathogenic HCoVs, such as Middle East respiratory syndrome-related coronavirus (MERS-CoV), SARS-CoV, and SARS-CoV-2 [13,14], represents an attractive model to deepen the study of substances already known for their anti-coronavirus activity in the search for more efficient antivirals.

Lactoferrin (Lf) is a multifunctional cationic iron-binding glycoprotein that plays a key role in fighting infections. This protein belongs to the transferrin family and is composed of two lobes (N- and C-lobes), each capable of reversibly chelating one Fe^+3^ ion [15]. The activity of bovine Lf (bLf) against infections by respiratory viruses such as RSV, adenovirus, IAV, and human parainfluenza virus type 2, has been extensively analyzed in in vitro systems [16,17,18,19,20,21,22,23]. Concerning coronaviruses, bLf has been demonstrated to be active against SARS-CoV [24] and SARS-CoV-2, HCoV-OC43, HCoV-NL63, and HCoV-229E [5,24,25].

Clinical trials on the efficacy of bLf against upper and lower respiratory infections [26,27], as well as SARS-CoV-2 [28,29], have also been performed. However, on the basis of contradictory results, further studies are needed.

Our previous studies demonstrated that bLf is able to bind to the IAV hemagglutinin (HA) of major virus subtypes such as H1N1 and H3N2 through its C-lobe fragments [30]. In particular, three fragments of the C-lobe, 418–429 (SKHSSLDCVLRP), 506–522 (AGDDQGLDKCVPNSKEK), and the modified sequence 552–563 (NGESSADWAKN), capable of inhibiting hemagglutination and infection at low concentrations, have been patented [31]. Subsequently, from the 418–429 fragment, through a truncation study, we identified two tetrapeptides, SLDC (peptide **1**) and SKHS (peptide **2**), capable of binding to HA and inhibiting viral infection at picomolar concentrations [32]. Starting from these sequences, we designed and synthesized a small library of N-methyl peptides and peptoids. Four compounds from this library demonstrated high-affinity binding to influenza A virus (IAV) HA and effectively inhibited hemagglutination and viral infection at picomolar concentrations [33]. It is known that RNA viruses have a higher mutation rate than DNA viruses, leading to the selection of viral strains resistant to antivirals, and the need to find new compounds to fight these infections is growing. Since the development of new drugs is a long and expensive process, numerous studies are focusing on the reuse of already-described compounds endowed with therapeutic properties. This approach, called drug/compound repurposing or repositioning, represents a valid emerging strategy in the field of drug discovery [34].

**Figure 1:**
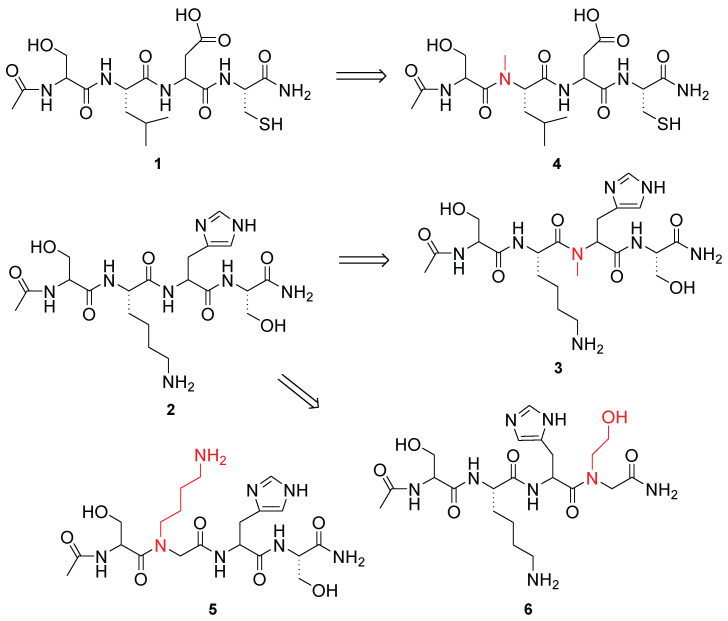
Structure of bLf-derived peptides and peptidomimetics tested in this study.

Based on these premises, in the present study we investigated whether bLf-derived peptides and/or peptidomimetics (Figure 1), previously identified and capable of inhibiting IAV infection, could also exert antiviral activity against coronaviruses.

By combining orthogonal biophysical analysis and biological and computational studies, we assessed the antiviral activity of the studied compounds and investigated their interaction with the HCov229E spike protein. Two bLf-derived tetrapeptides and two peptidomimetics able to inhibit coronavirus infection were identified. Our findings are encouraging since the most active peptidomimetics, SK(N-Me)HS (compound **3**) and S*N*KHS (compound **5**), have higher metabolic stability and better bioavailability than the tetrapeptide from which they are derived. The occurrence of new and drug-resistant viruses highlights the urgent need to rapidly develop novel antiviral strategies. This study confirms the importance and effectiveness of the drug repositioning approach as a viable strategy to combat viral respiratory tract infections.

## 2. Materials and Methods

### 2.1. Peptide Synthesis

Nα-Fmoc-protected amino acids, Fmoc Rink Amide AM resin, *N*, *N*-Diisopropylcarbodiimide (DIC), piperidine, and trifluoroacetic acid were purchased from Iris Biotech (Marktredwitz, Germany). Oxyma Pure was obtained from the CEM Corporation (Matthews, NC, USA). The peptidomimetic synthesis solvents and reagents, as well as CH_3_CN used for high-performance liquid chromatography (HPLC), were reagent-grade and were acquired from commercial sources and used without further purification unless otherwise noted.

Peptides **1** and **2** were synthesized previously [32].

#### 2.1.1. Synthesis of N-Methyl Peptides

The SPPS of the N-methyl peptides (**3** and **4**) was conducted following the Fmoc/tBu protocol using a Rink Amide AM resin (0.71 mmol/g) as the solid support. The synthesis was carried out on a Liberty Blue microwave automatic synthesizer (CEM Corporation, Matthews, NC). The standard couplings were made with Fmoc amino acids at a 0.2 M concentration and scale of 0.1, with DIC/OxymaPure^®^ used as the activator in DMF, at 90 °C, and the deprotection was performed with piperidine at a 30% concentration in DMF at 90 °C for 1 min. The coupling of Fmoc-N-Me-Leu-OH was performed at 75 °C for 5 min 2×. The coupling of Fmoc-N-Me-His (Trt)-OH was performed at 50 °C for 10 min. All the coupling cycles until the final deprotection were carried out with the aforementioned equipment. Following removal of the N-terminal Fmoc protecting group, the peptidomimetics were acetylated by treatment with Ac_2_O/DMF at 65 °C for 2 min. Finally, the peptide was released from the resin with TFA/iPr_3_SiH/H_2_O (90:5:5) for 2 h. The resin was removed by filtration, and the crude peptide was recovered by precipitation with cold anhydrous ethyl ether to yield a white powder and then lyophilized.

#### 2.1.2. Synthesis of Peptoids

The peptoids **5** and **6** were prepared at the 0.1 mmol scale using a CEM Liberty Blue automated microwave peptide synthesizer on Rink Amide AM resin (0.71 mmol/g). After resin deprotection with piperidine at a 30% concentration in DMF at 90 °C for 1 min, the standard couplings were made with Fmoc amino acids at a 0.2 M concentration and scale of 0.1, with DIC/OxymaPure^®^ used as the activator in DMF, at 90 °C for 2 min. For the peptoid residues, acylation was performed with 2 M bromoacetic acid and 2.4 M DIC at 75 °C for 5 min, and nucleophilic displacement was performed with 1 M monosubstituted amine in DMF at 75 °C for 5 min. All the coupling cycles until the final deprotection were carried out with the aforementioned equipment. After removal of the N-terminal Fmoc group, the peptoids were acetylated by treatment with an Ac_2_O/DMF solution at 65 °C for 2 min. Finally, the cleavage was performed as described above.

#### 2.1.3. Purification and Characterization

All the crude peptidomimetics were purified by RP-HPLC on a preparative C18-bonded silica column (Phenomenex, Luna Omega 100 Å, 100 × 21.20 mm, 5 µm) using a Shimadzu SPD 20A UV/VIS detector (Shimadzu Corporation, Kyoto, Japan), with detection at 214 and 254 nm. The column was perfused at a flow rate of 17 mL/min with solvent A (1% *v*/*v* water in 0.1% aqueous TFA), and the method adopted for peptidomimetic elution was an isocratic gradient: at 0.01–3.00 min, the solution contained 1% of solvent B (99% *v*/*v* acetonitrile in 0.1% aqueous TFA), then at 3.01–18.00 min, this increased to 1–20% of solvent B (80% *v*/*v* acetonitrile in 0.1% aqueous TFA), at 18.01–19.00 min, this increased to 90%, and at 19.01–20.00 min it returned to 1%. The analytical purity and retention time (tr) of each peptidomimetic was determined using the HPLC conditions in the above-mentioned solvent system (solvents A and B) programmed at a flow rate of 0.600 mL/min and fitted with a Phenomenex C-18 column and Luna Omega C18 column (100 mm × 2.10, 3 µm). The LC gradient was the following: at 0–7 min, there was 1–40% B; at 7.01–8 min, there was 40–90% B; and at 8.01–9 min, this returned to 1% B. All the peptidomimetic analogs exhibited a purity greater than 97% when monitored at 215 nm. Homogeneous fractions, as determined by analytical HPLC, were pooled and subsequently lyophilized. The molecular weights of the peptidomimetics were determined via positive-mode electrospray ionization (ESI) on an LTQ Orbitrap XL mass spectrometer (Thermo Scientific, Rheinfelden, Germany), using Xcalibur software (v3.0) for data acquisition and analysis. The samples were dissolved in a 1:1 (*v*/*v*) mixture of water and methanol and directly infused into the electrospray source using a syringe pump at a constant flow rate of 15 μL/min. Full analytical data are provided in the Supplementary Material (Table S1 and Figures S1–S5).

### 2.2. Biological Procedures

#### 2.2.1. Cells

MRC-5 cells (human lung fibroblasts, ATCC, CCL-171) were grown at 37 °C in an MEM (minimal essential medium) containing 10% FCS (fetal calf serum), L-glutamine (2 mM), sodium pyruvate (1 mM), nonessential amino acids (1%), and antibiotics (penicillin and streptomycin at 100 IU/mL and 100 μg/mL, respectively).

#### 2.2.2. Virus

Human coronavirus 229E (HCoV 229E, ATCC, VR-740) was grown by inoculating 80–90% confluent MRC-5 cells in 75 cm^2^ tissue culture flasks for 1 h in 1 mL of a medium. Subsequently, 20 mL of a fresh medium containing 2% FCS was added and the cells cultured for 3 to 5 days. The cytopathic effect (CPE) was monitored by light microscopy and the virus was harvested when 70% of the cells detached due to the CPE. The supernatant was centrifuged for 10 min at 3000 rpm, aliquoted, and stored at −80 °C.

#### 2.2.3. Virus Titration

MRC-5 cells cultured in 96-well microplates (1.7 × 10^4^ cells/well) were infected with serial 10-fold dilutions of the HCoV 229E stock (100 μL/well) for 1 h at 37 °C. After viral adsorption, the infected and mock-infected cells were rinsed thoroughly and incubated with a fresh medium containing 2% FCS at 37 °C under a humidified atmosphere of 5% CO_2_ for 72 h. The CPE was calculated from the average of at least 6 replicates. Wells with a cytopathic effect were scored as positive for virus growth, and the median tissue culture infectious dose (TCID_50_) was determined using Reed and Muench’s method [35].

#### 2.2.4. MTT (3-[4,5-Dimethyl-2-thiazolyl]-2,5-diphenyl-2htetrazolium Bromide) Assay of Cell Viability

This procedure was performed as reported elsewhere [36]. MRC-5 cells, cultured in 96-well microplates, were treated at 37 °C in a humidified atmosphere of 5% CO_2_ with two-fold serial compound dilutions in MEM.

After 1 h, the cells were washed and incubated with a 2% FCS medium containing the compounds for 72 h at 37 °C. Every 24 h the cell morphology was evaluated by light microscope observation. After 72 h, the cells were washed in phosphate-buffered saline (PBS, pH 7.4), and 100 μL of an MTT solution (0.5 mg/mL in PBS) was added to each well. After 3 h of incubation at 37 °C, the liquid was removed, and formazan crystals were dissolved in 100 μL of dimethyl sulfoxide (DMSO). After 15 min of incubation at room temperature, the microplates were read using an ELISA plate reader (PerkinElmer Italia, Monza, Italy) with a 570 nm test wavelength and a 690 nm reference wavelength. The cytotoxicity was calculated from the average of at least 6 replicates. Compound concentrations not affecting the cell vitality compared to the untreated cells were used for the antiviral tests.

#### 2.2.5. Antiviral Assay

Cells were grown in 96-well microplates at 37 °C in 5% CO_2_. After 24 h an antiviral assay was carried out by incubating serial compound dilutions (starting from 25 μM) with equal volumes of viral suspensions for 1 h at 4 °C. Viral suspensions incubated with the medium alone were utilized as control viruses. The cells were then infected with 100 μL/well of the virus–compound mixtures at 37 °C. After 1 h, the infected cells were washed and incubated with a 2% FCS medium containing the compounds for 72 h at 37 °C. To assess the effect on the early stages of infection, other experiments were conducted in which the compounds were only present during either the viral adsorption phase (1 h) or during the adsorption and then added again for the entire viral replication cycle (1 h + 72 h). The CPE was measured using an MTT assay as already described. The results were expressed as the percentage of CPE inhibition in comparison with that of the untreated control cultures. The following formula was used to determine the protection percentage: (A_570_ compound-virus − A_570_ virus)/(A_570_ mock infected cells − A_570_ virus) × 100%. The CPE was calculated from the average of at least 6 replicates, and three independent experiments were performed. EC_50_ and EC_25_ indicate the concentration of the compound that inhibited infection by 50% and 25%, respectively.

### 2.3. Direct Binding Assay

The HCoV-229E spike protein was purchased from Acrobiosystems (Cat. No. SPN-H52H3). CM5 sensor chips, an HBS-P+ buffer (0.01 M HEPES, pH 7.4, 0.15 M NaCl, 0.05% *v*/*v* Surfactant P20), 1-ethyl-3-(3-dimethylaminopropyl)carbodiimide hydrochloride (EDC), N-hydroxysuccinimide (NHS), ethanolamine (H_2_N(CH_2_)_2_OH), and a regeneration solution were obtained from Cytiva.

#### 2.3.1. Microscale Thermophoresis (MST)

MST experiments were performed using a Monolith NT.115pico instrument (NanoTemper Technologies, Munich, Germany). The HCoV-229E spike protein was labeled using a RED-tris-NTA 2nd Generation His-Tag Labeling Kit (NanoTemper Technologies) according to the manufacturer’s instructions. Briefly, 100 μL of the HCoV-229E spike protein (80 nM in double-distilled water) was mixed with 100 μL of 40 nM NT647-NHS fluorophore in a labeling buffer and incubated at room temperature for 30 min. Following incubation, the mixture was centrifuged at 15,000× *g* for 10 min at 4 °C to remove aggregates.

Preliminary tests were conducted using both standard-treated and premium-coated MST capillaries (NanoTemper Technologies) as described previously [37]. No significant protein adsorption to standard-treated capillaries was observed in phosphate-buffered saline (PBS), which was therefore selected as the assay buffer for subsequent experiments.

To ensure optimal MST signal repeatability and minimize non-specific adsorption to the capillary walls, the buffer conditions were evaluated. Compound stock solutions (5 mM) were diluted in the MST buffer to a maximum soluble concentration of 50 μM. For the binding assays, 16-point, 1:1 serial dilutions of each compound were prepared and mixed with the NT647-labeled HCoV-229E spike protein, with a final reaction volume of 20 μL. After a 30 min incubation at rt, the samples were loaded into standard-treated capillaries and placed in a Monolith NT.115 chip tray for thermophoresis analysis.

Fluorescence signals were recorded using high MST power and 10% LED power. Dissociation constants (K_D_) were determined from the concentration-dependent changes in the normalized fluorescence (F_norm_) measured 21 s after thermophoresis initiation. Each compound was tested in triplicate, and the data were analyzed using MO Affinity Analysis software (v2.3, NanoTemper Technologies, Munich, Germany). The K_D_ values are reported with the associated confidence intervals (±) for each tested compound.

#### 2.3.2. Surface Plasmon Resonance (SPR)

SPR experiments were conducted at 25 °C using a Biacore T200 instrument (Cytiva, Marlborough, UK). The HCoV-229E spike protein (0.16 μM in 10 mM sodium acetate, pH 4.0) was injected over a CM5 sensor chip surface and covalently immobilized via standard amine coupling to a final level of approximately 3500 response units (RUs). No protein was immobilized on the reference flow cell. Following immobilization, the chip was stabilized by injecting an HBS-P+ running buffer (0.01 M HEPES, pH 7.4, 0.15 M NaCl, 0.05% *v*/*v* Tween 20) overnight at a flow rate of 5 μL/min.

For the binding assays, peptidomimetics solutions at various concentrations were prepared in an HBS-P+ buffer and injected over the immobilized spike protein at 25 °C with a flow rate of 30 μL/min. The association phase was monitored for 120 s, followed by dissociation in the buffer alone for 300 s. Dissociation of the spike protein during immobilization was monitored by injecting a running buffer for 400 s immediately after coupling. The global fitting of a 1:1 binding model in BIAevaluation software (v3.1) was used for determining the equilibrium dissociation constants (*K_D_*), and the kinetic dissociation (*k_off_*) and association (*k_on_*) constants were calculated using Equations (1) and (2).

(1) $$\frac{dR}{dt}=k_{on\;}\times C\times \left(R_{max}-R\right)-k_{off\;\;}\times R$$

where *R* represents the response unit, *C* is the concentration of the analyte, and

(2) $$K_{D}=\frac{k_{off}}{k_{on}}$$

#### 2.3.3. Nanoscale Differential Scanning Fluorometry

The assay was performed in a PBS buffer (pH 7.4) using 2 μM of the HCoV-229E spike protein and 200 μM of each compound. The samples were loaded into nanoDSF Grade Standard Capillaries (NanoTemper Technologies) and analyzed using a Prometheus NT.48 nanoDSF device (NanoTemper Technologies). The protein thermal unfolding was monitored using a linear thermal ramp (1 °C/min) from 20 °C to 95 °C, with an excitation power of 70% (Tables S2 and S3).

The peptidomimetics’ binding affinity was estimated from the fluorescence intensity ratio at 330 nm and 350 nm, measured at 20 °C and 95 °C to represent the native and unfolded states of the protein, respectively (Figures S6–S13). For the control, the compound was replaced with a buffer. Each compound was tested in triplicate, and the average results are reported. The fluorescence intensity ratios and their first derivatives were calculated using the manufacturer’s software (PR.ThermControl, version 2.1.2).

### 2.4. Computational Studies

The in silico studies were performed using the Schrodinger Suite, version 2025-1 [38] Schrodinger 2025-1].

The ligand peptidomimetics were drawn as 2D structures and optimized using LigPrep [38] to obtain the correct protonation state at a physiological pH. The resulting ligand structures were minimized using MacroModel [39], applying the OPLS4 force field, the GB/SA water solvation model, and the PRCG algorithm to achieve convergence to 0.01 kJÅ^−1^ mol^−1^.

The HCoV229E spike protein structures available in the Brookhaven Protein Data Bank (https://www.rcsb.org/) (accessed on 27 October 2024) at the time of the study (PDB IDs: 6U7H, 7CYC, and 7CYD) were obtained by cryo-EM at a low resolution (3.1, 3.21, and 3.55 Å, respectively). The protein structures were prepared using the protein preparation routine available in Maestro [38], which adds hydrogen atoms, determines the protonation states and preferred tautomers, and minimizes the proteins.

SiteMap [40,41] was used to identify the most promising binding sites on the available S proteins by computing a fine grid and saving a maximum of 20 sites per structure. The best scoring sites were reviewed to remove duplicates and inaccessible sites. A total of eleven sites were selected for subsequent docking studies. The docking grids were defined using the molecular interaction fields generated by SiteMap. Docking calculations were carried out using the Glide SP-Peptide protocol. For each ligand, up to 100 poses were generated and saved [42]. The best scoring sites were analyzed and the most promising was selected considering the docking scores and the reproducibility of the docking poses.

## 3. Results

Since the development of new drugs is a long and expensive process, in this work we adopted the compound repurposing approach as a suitable strategy to provide a rapid response to the emergency of pandemic viruses. To evaluate the potential antiviral activity of bLf-derived peptides and peptidomimetics against coronaviruses, we conducted a series of in vitro experiments using HCoV-229E as a model system. A series of biophysical assays verified the interaction of the studied compounds with the HCov229E spike protein, while the structure-based computational studies provided a possible explanation of the mechanism of action.

Here, we present the results of these experiments, which highlight differences in efficacy among the tested compounds and confirm the potential of specific peptidomimetics for use as broad-spectrum antiviral agents.

### 3.1. Antiviral Assay

The ability of compounds **1**–**6** to inhibit viral infection was evaluated. The results obtained were compared to those previously obtained for IAV. Only two compounds, **3** and **5**, were able to prevent coronavirus infection in a dose range of roughly 0.031 to 0.0177 nM, corresponding to selectivity indices ≈ 10^5^/10^6^ (Table 1).

To evaluate a possible effect in the early stages of HCoV-229E infection, experiments were carried out in which the compounds were present in different concentrations only during the viral adsorption step or throughout the infection (Table 2).

The results of these experiments showed an antiviral effect of the two compounds in the initial phases of viral infection, such as attachment, internalization, or membrane fusion (uncoating), although with some differences between the compounds. In fact, the activity of compound **3** was higher when it was present during the entire cycle of the infection, i.e., attachment and replication, as at a concentration of 4.3 × 10^−2^ nM it was able to inhibit attachment by 25% and the entire infection cycle by 50%. Compound **5** essentially exerted its inhibitory action in the early phases of viral infection, such as attachment, internalization, or uncoating, as the degree of inhibition achieved in this condition was almost the same as that achieved when the compound was present during the entire cycle of infection (EC50 of 2.0 ± 0.2 × 10^−2^ nM and EC50 of 2.5 ± 0.4 × 10^−2^ nM, respectively).

### 3.2. Biophysical Characterization of Spike-Binding Peptidomimetics

Biophysical techniques are highly valuable for conducting detailed analyses of protein–ligand interactions [43]. An effective strategy for hit validation involves integrating multiple methods. In this study, we investigated the binding affinity using three complementary techniques: MST, SPR, and DSF (Table 3).

#### 3.2.1. Peptidomimetic–Spike Interactions Determined by MST

MST is a technique used to quantify the binding affinity between peptides and proteins by measuring the movement of fluorescently labeled molecules across a microscopic temperature gradient. The binding of a ligand to a protein alters its thermophoretic behavior, allowing for the determination of its binding affinity in solution. By monitoring the fluorescence signal as a function of the ligand concentration, MST allows for precise determination of the dissociation constant (K_D_) in solution under near-native conditions.

**Figure 2:**
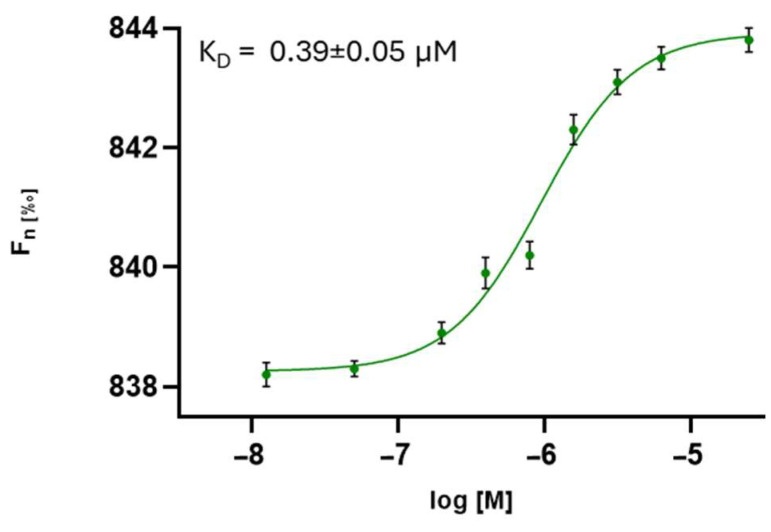
Direct measurements of the binding of compound **3** to HCoV-229E S protein. The dose–response curve for the interaction between compound **3** and the HCoV-229E S protein is shown, with F_norm_ representing the normalized fluorescence. Experiments were independently repeated three times. The reported K_D_ value represents the mean ± the standard deviation (SD) of these three independent measurements.

The compounds were screened by MST, as detailed in the Materials and Methods (Section 2), and the results are reported in Table 3. The analysis revealed that all the compounds interacted with the HCoV-229E S protein with different dissociation constants. In particular, peptidomimetic **3** effectively bound the HCoV-229E spike protein (Figure 2).

#### 3.2.2. Binding Affinity of Compounds to Spike Protein Determined by SPR

SPR is a label-free technique that monitors the real-time interactions between immobilized proteins and ligands in solution. The target protein is fixed onto a thin metal film, and ligand binding induces changes in the refractive index at the sensor surface. These changes alter the angle of the reflected light, producing a signal proportional to the amount of the bound ligand. By recording the association and dissociation phases, SPR provides detailed kinetic data and affinity constants for the protein–ligand interaction.

Up to about 3500 response units (RUs) of HCoV-229E S protein (His-Tag) was immobilized on a sensor chip for the SPR study (see the Materials and Methods, Section 2). Upon injection of the test compounds, binding to the immobilized protein was detected as a change in the refractive index at the sensor surface [44]. Following injection, a continuous flow of a running buffer was applied to monitor the dissociation phase. No HCoV-229E S protein was immobilized on the reference (control) flow cell, which was used to subtract the non-specific binding and bulk refractive index changes. As expected, no appreciable signal was detected in the control channel.

**Figure 3:**
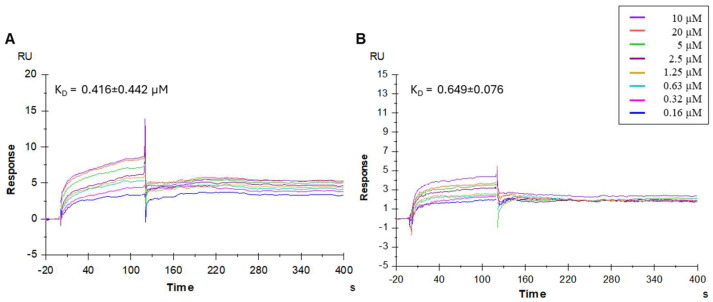
Surface plasmon resonance (SPR) analysis. Sensorgrams showing the binding affinity between the HCoV-229E spike protein and compounds **3** (**A**) and **5** (**B**). Each compound was injected at eight different concentrations (0.16, 0.32, 0.63, 1.25, 2.5, 5, 10, and 20 µM). The equilibrium dissociation constants (K_D_) were calculated from the ratio of the kinetic dissociation (k_off_) and association (k_on_) rate constants.

The SPR study demonstrated that the synthesized peptidomimetics effectively interacted with the immobilized HCoV-229E S protein. The sensorgrams of compounds **3** and **5** bound to the HCoV-229E S protein in an HBS-P+ buffer are shown in Figure 3. Notably, these peptidomimetics bound the HCoV-229E spike protein with high efficiency.

#### 3.2.3. NanoDSF of Spike Protein–Peptidomimetics

NanoDSF is an advanced form of differential scanning fluorimetry used to measure the melting temperature (Tm) of peptidomimetic–protein complexes. In this technique, a protein in solution is subjected to a controlled temperature gradient, leading to its thermal unfolding. The intrinsic fluorescence of the protein, primarily arising from aromatic side chains of tyrosine and tryptophan residues, is monitored throughout the process. As the protein unfolds, these residues become exposed to the solvent, resulting in measurable changes in the fluorescence intensity. The correlation between the fluorescence changes and temperature allows for the determination of the apparent Tm.

All the compounds were screened at a concentration of 200 μM using nanoDSF. The compounds exhibited thermal shifts (ΔTm) ranging from −0.9 °C to 0.9 °C. In particular, those showing a ΔTm ≥ 0.3 °C (used as the selection threshold) were considered potential inhibitors. Based on this criterion, compounds **3** and **5** were identified as hits, showing ΔTm values ranging from 0.3 to 0.9 °C at 200 μM (Table 3). The experiments were repeated independently twice.

### 3.3. Computational Studies

The results of the antiviral studies show the effective activity of compounds **3** and **5** against the HCoV229E infection and suggest the action of compound **5** in the early stages of infection. On the other hand, the biophysical assays demonstrated effective binding of compound **5** with the spike protein, suggesting that its antiviral activity could be due to its interaction with this viral receptor. Therefore, the computational analysis was aimed at simulating the interaction between the studied compounds and the protein responsible for binding to the host cell. Common to multiple viruses, the S protein is responsible for both host cell binding and membrane fusion [45]. Host receptor binding occurs in the receptor-binding domain (RBD) located in the N-terminal S1 subunit, while the fusion peptide is located in the C-terminal S2 subunit. In particular, the S1 RBD subunit is exposed to contact with APN.

**Figure 4:**
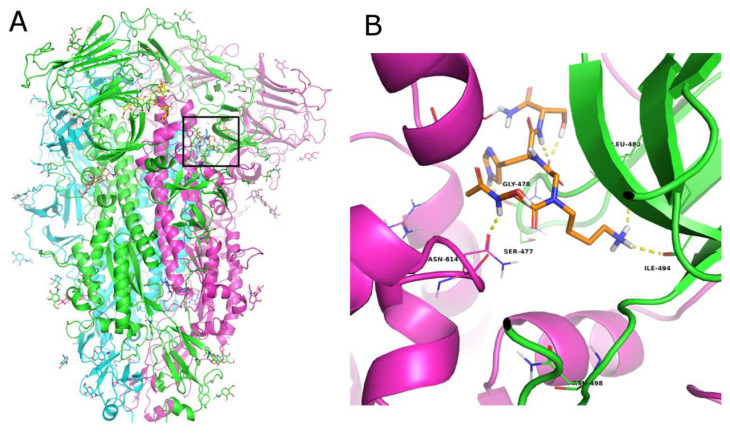
(**A**) Cartoon representation of the trimeric structure of the HCoV229E spike protein. Each monomer is colored differently. The binding site is marked with a black square. Zoomed-in view of the docked poses of ligand **5** ((**B**), orange C atoms) in the S protein. The protein is shown as a cartoon, the interacting residues are represented as lines, and the H-bonds are depicted as dashed yellow lines.

Three structures of the HCov229E S protein were available in the Protein Data Bank at the time of this study (PDB IDs: 6U7H [46], 7CYC, and 7CYD [47]. All the available structures were obtained by cryo-EM with a low resolution (range of 3.1–3.55 Å) and were used for ensemble structure-based computational studies as they described different conformations of the spike protein in a pre-fusion state. Since there were no experimental data on the binding site for small molecules, a SiteMap [38] analysis was performed [40,41], focusing on the N-terminal region of the S protein. A total of eleven sites presenting the best D-score were selected in the three structures, after eliminating sites located in the inaccessible inner part of the protein or replicated in the trimeric structure (Table S4 and Figure S14). The active compounds were docked into each of the eleven sites using an SP peptide docking approach that explored the high conformational flexibility of the ligands [33,48]. The active ligand that obtained the highest docking score in the 7CYC structure (Table S5) corresponded to activated conformation 1 in the pre-fusion state between subdomain 1 (SD1) of one monomer and the S2 subunit of another monomer [47]. In the docked complexes, ligand **5** interacted with Ser477, Gly478, Asn479, Leu480, and Ile494 of chain A and Asn614 and Gln863 of chain C (Figure 4). These interactions of the ligand with residues of different chains in the spike suggest possible interference with the conformational reorganization of the S protein necessary to complete the fusion process.

## 4. Discussion

In the present study we expanded our antiviral research focusing on a virus involved in the common cold, namely HCoV229E, that could also represent a model for the more threatening coronaviruses. HCoV-229E, first isolated in 1966 [49], is responsible for the common cold in healthy adults but can cause lower respiratory tract infections in young children and the elderly. This virus can cause severe and life-threatening infections in immunocompromised patients. It is also important to highlight that HCoV-229E infection is not always limited to the respiratory tract and the virus can invade the central nervous system (CNS). RNA from HCoV-229E and HCoV-OC43 have in fact been detected in human brain samples, demonstrating that these respiratory pathogens are neuroinvasive and can establish a persistent infection in the human CNS [13]. It has been demonstrated that the human aminopeptidase N (hAPN/CD13) receptor, expressed on neuronal and glial cells, facilitates HCoV-229E entry and infection, further supporting its neurotropic potential [50]. In addition, the possible involvement of HCoV-229E in the development of Kawasaki disease has been hypothesized [51].

Currently, there are no approved antiviral drugs or vaccines that have been specifically developed for HCoV-229E, but several strategies are being developed to effectively control this and other respiratory viruses. These strategies, in addition to the use of potential vaccines, focus on the inhibition of viral replication using drugs. However, as future coronavirus epidemics are highly possible, it is important to develop broad-spectrum antivirals capable of preventing and treating infections by both the currently circulating CoVs and CoVs emerging in this future. In this context, creating novel anti-coronavirus treatments for all highly pathogenic HCoVs remains a significant challenge.

In this study, we employed a compound repurposing strategy to identify potential inhibitors targeting the HCoV-229E virus by focusing on bLF-derived peptides and related peptidomimetics already shown to prevent the early phases of IAV infection.

In vitro antiviral assays showed that the two tetrapeptides (**1**–**2**) derived from the 418–429 fragment of the bLf C-lobe are able to inhibit coronavirus infection, although less effectively than IAV infection. On the other hand, two of the four peptidomimetics (**3**–**6**) endowed with anti-influenza activity were shown to inhibit coronavirus infection more effectively than IAV infection. Compounds **3** and **5**, in fact, were found to be potent and selective inhibitors of HCoV-229E, displaying EC_50_ values in the subnanomolar range (31 and 17.7 pM, respectively) with high selectivity indices (>10^5^). These potency levels far exceed those reported for fusion-inhibitory peptides targeting HCoV-229E spike HR regions, such as 229E-HR2P, which exhibited IC_50_ ≈ 0.3–1.7 µM in cell-based assays [52].

Functional studies on the stages of viral infection revealed that compound **3** inhibits both the early and late phases of viral infection, while compound **5** primarily targets early infection events such as virus attachment and penetration. This mode of action is comparable to that reported for lactoferrin-derived peptides, which inhibit coronavirus entry by competing with viral particles for host cell surface components, particularly heparan sulfate proteoglycans [53,54]. However, while these natural peptides exhibited antiviral activity in the micromolar range (IC_50_ > 1 µM), compounds **3** and **5** demonstrated over 10^3^-fold higher potency, with subnanomolar EC_50_ values and superior selectivity indices.

The integration of multiple biophysical techniques—Microscale Thermophoresis (MST), surface plasmon resonance (SPR), and Nano Differential Scanning Fluorometry (nanoDSF)—allowed for a comprehensive characterization of the binding affinity between the compounds and the target protein.

The MST results highlighted compound **3** (SK(N-Me)HS) as exhibiting the highest binding affinity to the S protein, with a K_D_ in the submicromolar range (0.39 µM), confirming a strong and specific interaction (Figure 1). These findings were supported by SPR analysis, which revealed consistent kinetic profiles and effective binding for compounds **3** and **5**, reinforcing the MST data. NanoDSF further corroborated these interactions, demonstrating the significant thermal stabilization of the spike protein, especially in the presence of compound **5** (ΔTm = 0.9 °C), suggesting ligand-induced conformational stabilization.

SK(N-Me)HS (**3**)and S*N*KHS (**5**) are two structurally modified peptidomimetics derived from the SKHS tetrapeptide, each bearing distinct chemical modifications aimed at improving their pharmacological properties. In SK(N-Me)HS (**3**), the nitrogen atom of the histidine residue is N-methylated, a modification that increases its steric hindrance and reduces its hydrogen bonding capacity and conformational flexibility. This alteration can enhance its metabolic stability by making the peptide less susceptible to proteolytic cleavage and may improve its membrane permeability [55,56].

S*N*KHS (**5**) features a peptoid-like modification in which the side chain of lysine is shifted from the ε-position to the α-nitrogen, effectively replacing the α-hydrogen with a side chain-bearing nitrogen. This structural change also increases the resistance to proteolytic degradation and alters the conformational flexibility of the molecule, potentially affecting its binding affinity and specificity.

These two modifications represent different strategies to optimize the parent peptide: SK(N-Me)HS (**3**) focuses on improving the stability through backbone modification, while S*N*KHS (**5**) introduces a structural rearrangement that mimics natural side chain functionality but avoids enzymatic recognition.

Interestingly, despite their distinct structural modifications, both SK(N-Me)HS (**3**) and S*N*KHS (**5**) demonstrated effective interaction with the spike protein of HCoV-229E. This suggests that the chemical alterations introduced in these peptidomimetics do not impair, and may even enhance, their ability to recognize and bind key viral surface structures.

Computational modeling provided a molecular basis for these observations by simulating the binding of compound **5** to the S protein. The HCoV229E spike is a large trimeric protein that undergoes significant conformational rearrangement to bind its host receptor, aminopeptidase N (APN) [57]. The 3D structures used in this study represented three different pre-fusion conformations of the S protein, all of which were considered. We explored the surface of the spike protein to find druggable binding sites. Docking studies identified the most promising binding region, which is located at the interface between the S1 and S2 subunits of the S trimer. Interestingly, the identified site corresponds to the one explored by Wang et al. in the SARS-CoV-2 spike protein in order to identify allosteric binders that can stabilize the ‘down’ conformation of the S protein. This is achieved by connecting the SD1 domain with the S2 subunit, thereby hindering conformational change in the receptor-binding domain (RBD) [58,59]. These results suggest that compound 5 may exert its antiviral activity by interfering with the conformational rearrangements of the spike protein required for membrane fusion, by binding to this inter-domain allosteric site rather than to the RBD, which is subject to a high frequency of mutations. This aligns with the observed inhibition in the early infection stages and biophysical screening.

The N-methylation of histidine in SK(N-Me)HS likely increases the metabolic stability of the peptide and the membrane permeability without disrupting the spatial orientation of functional groups critical for spike protein binding. Similarly, the repositioning of the lysine side chain in S*N*KHS, which converts it into a peptoid-like structure, may preserve or even optimize the spatial presentation of key residues involved in the interaction with the viral spike.

These findings indicate that both modifications retain the molecular determinants necessary for effective binding to the HCoV-229E spike protein, supporting their potential use as lead compounds for the development of novel coronavirus entry inhibitors.

Overall, these results support the use of compounds **3** and **5** as promising antiviral candidates against HCoV-229E, and potentially other coronaviruses, due to their high binding affinity and potent antiviral activity. Future studies are necessary to experimentally verify the proposed mechanism of action and the binding of the S protein. Furthermore, additional investigations should focus on these compounds’ pharmacokinetics, in vivo stability, and efficacy against other coronaviruses.

## 5. Conclusions

In conclusion, this study successfully identified two peptidomimetic compounds, **3** and **5**, that were potent inhibitors of the HCoV-229E coronavirus. Their strong binding affinity to the spike protein, confirmed using complementary biophysical techniques, correlated with their high antiviral efficacy in vitro. Computational analyses suggested possible interference with conformational changes in the spike protein necessary for viral entry and fusion. These findings highlight the potential of these molecules as promising candidates for further development as antiviral agents against HCoV-229E and possibly other related coronaviruses. Future investigations will be essential to assess their pharmacological properties and antiviral spectrum in vivo.

## Figures and Tables

**Table 1:** Sequence and antiviral activity of peptides **1** and **2** and peptomimetics **3**–**6**.

Compound	Sequence	Influenza A H1N1 Virus ^a,b^		229E Coronavirus	
		EC_50_^c^ (nM)	SI ^d^	EC_50_^c^ (nM)	SI ^d^
**1**	SLDC	4.6 ± 0.05 × 10^−3^	>5.4 × 10^6^	50 ± 0.4	>5 × 10^2^
**2**	SKHS	4.8 ± 0.12 × 10^−5^	>5.2 × 10^8^	500 ± 2	>5 × 10^1^
**3**	SK(N-Me)HS	0.142 ± 0.002	>1.78 × 10^3^	0.031 ± 0.007	>8 × 10^5^
**4**	S(N-Me)LDC	0.085 ± 0.0004	>2.9 × 10^5^	--	--
**5**	S*N*KHS ^e^	0.470 ± 0.002	>5.3 × 10^4^	0.0177 ± 0.002	>14.1 × 10^5^
**6**	SKH*N*hS ^e^	0.500 ± 0.004	>5 × 10^5^	--	--

^a^ [32]. ^b^ [33].^c^ EC_50_: the compound concentration that reduced the cytopathic effect by 50%; ^d^ SI: the ratio of CC_50_ (the compound concentration that reduced the cell proliferation by 50%, typically > 25 μM) to EC_50_. The mean values of three independent experiments with their standard errors are shown. ^e^ Abbreviations: *N*K: *N*-(4-aminobutyl)glycine; *N*hS: *N*-(2-hydroxyethyl)glycine.

**Table 2:** Antiviral activity of peptomimetics in different phases of infection.

Compound	Sequence	Adsorption	Infection Cycle
**3**	SK(N-Me)HS	EC_25_^a^ (nM) 4.3 ± 0.6 × 10^−2^	EC_50_^b^ (nM) 4.3 ± 0.6 × 10^−2^
**5**	S*N*KHS	EC_50_^b^ (nM) 2.5 ± 0.4 × 10^−2^	EC_50_^b^ (nM) 2.0 ± 0.2 × 10^−2^

^a^ EC_25_: the peptidomimetic concentration that reduced the cytopathic effect by 25%. ^b^ EC_50_: the peptidomimetic concentration that reduced the cytopathic effect by 50%.

**Table 3:** Sequences of and binding affinities between HCoV-229E spike protein and compounds **3**–**6** determined using MST, SPR, and DSF.

Compound	Sequence ^a^	MST K_D_ (µM)	SPR K_D_ (µM)	∆Tm (°C)
**1**	SLDC	4.92 ± 0.7	>50	−0.9
**2**	SKHS	5.12 ± 0.6	41.44 ± 0.87	−0.7
**3**	SK(N-Me)HS	0.39 ± 0.05	0.416 ± 0.442	0.3
**4**	S(N-Me)LDC	2.6 ± 1.3	1.153 ± 0.182	0.1
**5**	S*N*KHS	3.3 ± 1.7	0.649 ± 0.076	0.9
**6**	SKH*N*hS	11 ± 0.8	1.507 ± 0.465	0.4

^a^ All compounds were amidated at C-terminal and acetylated at N-terminal.

## Data Availability

The data are presented within the article and the Supplementary Materials.

## Supplementary Materials

The following supporting information can be downloaded at: https://www.mdpi.com/article/10.3390/pharmaceutics17081006/s1, Table S1: Analytical data of peptides **1**–**6**; Figures S1–S4: HRMS spectra and HPLC chromatograms of peptide **3**–**6**; Figures S5–S7: MST binding curves of peptides **1**, **2**, **4**–**6**; Table S2: NanoDSF assays HCoV-229E-peptide **1**–**2**; Figures S8–S10: Melt curves and the first derivative of the melt curves; Table S3: NanoDSF assays HCoV-229E-peptide **3**–**6**; Figures S11–S14: Melt curves and the first derivative of the melt curves; Table S4: Calculated SiteMap scores; Figure S15: Docked poses of S protein; Table S5: Docking score values compounds **5**-S protein.
