# Supplementary material for: Inhibition of Human Coronavirus 229E by Lactoferrin-Derived Peptidomimetics
Source: Pharmaceutics. 2025 Aug 1;17(8):1006. doi: 10.3390/pharmaceutics17081006 (PMC12389581; doi:10.3390/pharmaceutics17081006)
Supplement: Supplementary file 1 [file pharmaceutics-17-01006-s001.zip › pharmaceutics-3769272-supplementary.pdf]

SUPPLEMENTARY MATERIALS

Inhibition of human coronavirus 229E by lactoferrin-derived peptidomimetics

Maria Carmina Scala [1], Magda Marchetti [2], Martina Landi[1], Marialuigia Fantacuzzi[3], Fabiana Superti[4,*], Mariangela Agamennone[3], Pietro Campiglia [1] and Marina Sala [1,*]

[1] Department of Pharmacy, University of Salerno, Via Giovanni Paolo II 132, 84084 Fisciano, Italy; mscala@unisa.it (M.C.S.); martlandi@unisa.it (M.L.); pcampiglia@unisa.it (P.C.)

[2] National Centre for Innovative Technologies in Public Health, National Institute of Health, Viale Regina Elena, 299, 00161 Rome, Italy; magda.marchetti@iss.it (M.M.)

[3] Department of Pharmacy, University "G. d'Annunzio" of Chieti-Pescara, Via dei Vestini 31, 66100 Chieti, Italy; marialuigia.fantacuzzi@unich.it (M.F.); magamennone@unich.it (M.A.)

[4] Association for Research on Integrative Oncology Therapies (ARTOI) Foundation, Via Ludovico Micara, 73, 00165 Rome, Italy; fabiana.superti@artoi.it (F.S.)

* Corresponding author: msala@unisa.it; fabiana.superti@artoi.it

Table of contents:

Supplementary schemes and figures:

**Table S1:**	Analytical data of peptides **1-6**	S1
**Figure S1-S4:**	HRMS spectra and HPLC chromatograms of peptide **3-6**	S2
**Figure S5-S7:**	MST binding curves of peptides **1,2, 4-6**........	S6
**Table S2**	NanoDSF assays HCoV-229E-peptide **1-2**	S7
**Figure S8-S10**	Melt curves and the first derivative of the melt curves	S8
**Table S3**	NanoDSF assays HCoV-229E-peptide **3-6**	S9
**Figure S11-S14**	Melt curves and the first derivative of the melt curves	S10
**Table S4**	Calculated SiteMap scores	S13
**Figure S15**	Docked poses of S protein	S14
**Table S5**	Docking score values compounds **5**-S protein	S14

Table S1. Analytical data of peptides **1**-**6**.

**Pep.**	**Sequence**	**HPLC k′[a]**	**HRMS**
**1**[b]	SLDC	3.93	477.18970
**2**[b]	SKHS	4.68	499.26235
**3**	S(N-Me)LDC	5.25	492.2395
**4**	SK(N-Me)HS	1.75	514.1417
**5**	S*N*KHS	3.72	499.3402
**6**	SKH*N*hS	3.61	514.3417

[a] k′=[(peptide retention time-solvent retention time)/solvent retention time]; [b] Scala et al. (2017).

Supplementary figures of Mass spectrometry and HPLC of peptides used in the study

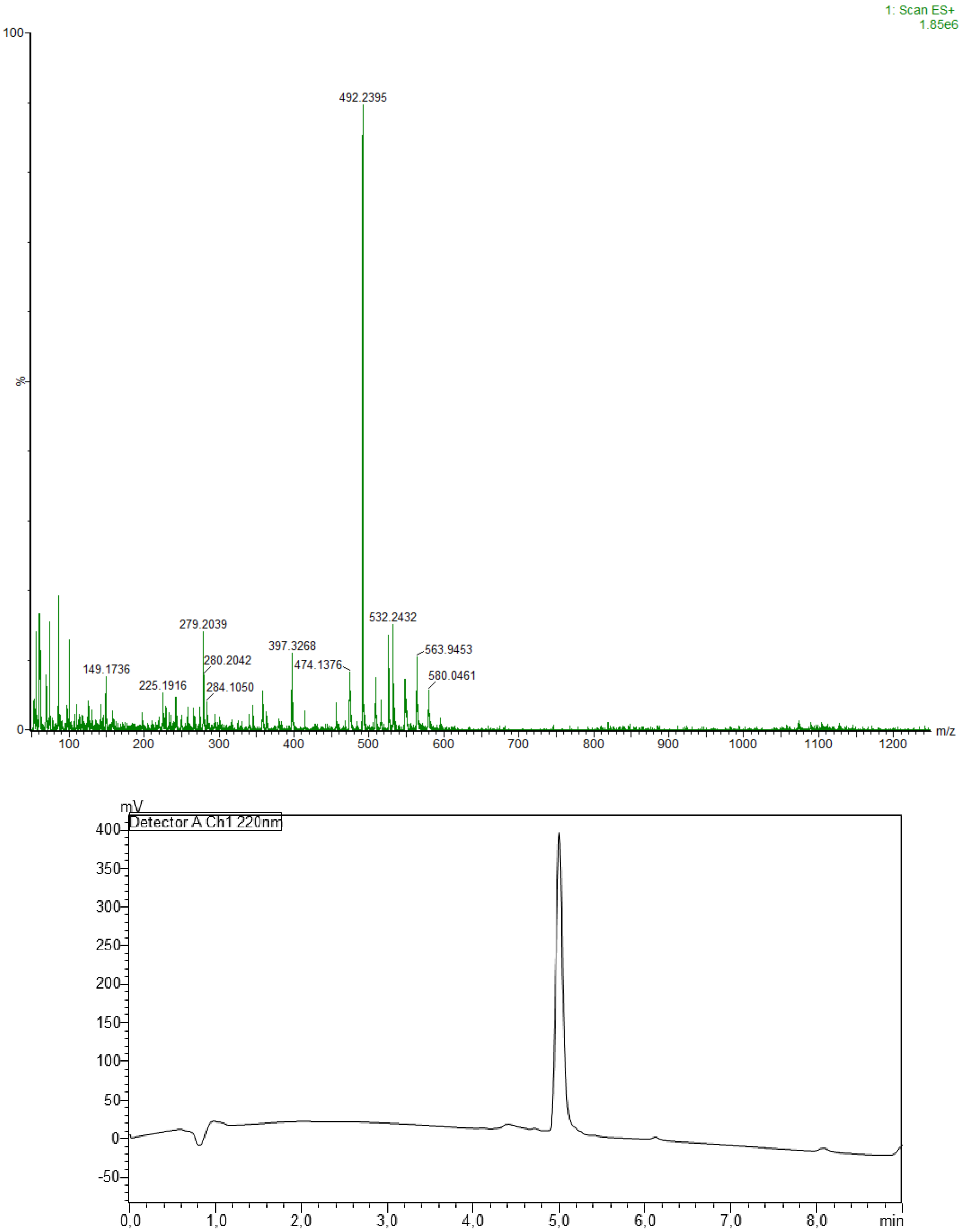

Figure S1. HR-ESI-MS of Peptide **3** ion [M+H]$^+$ and analytical HPLC trace at 220 nm.

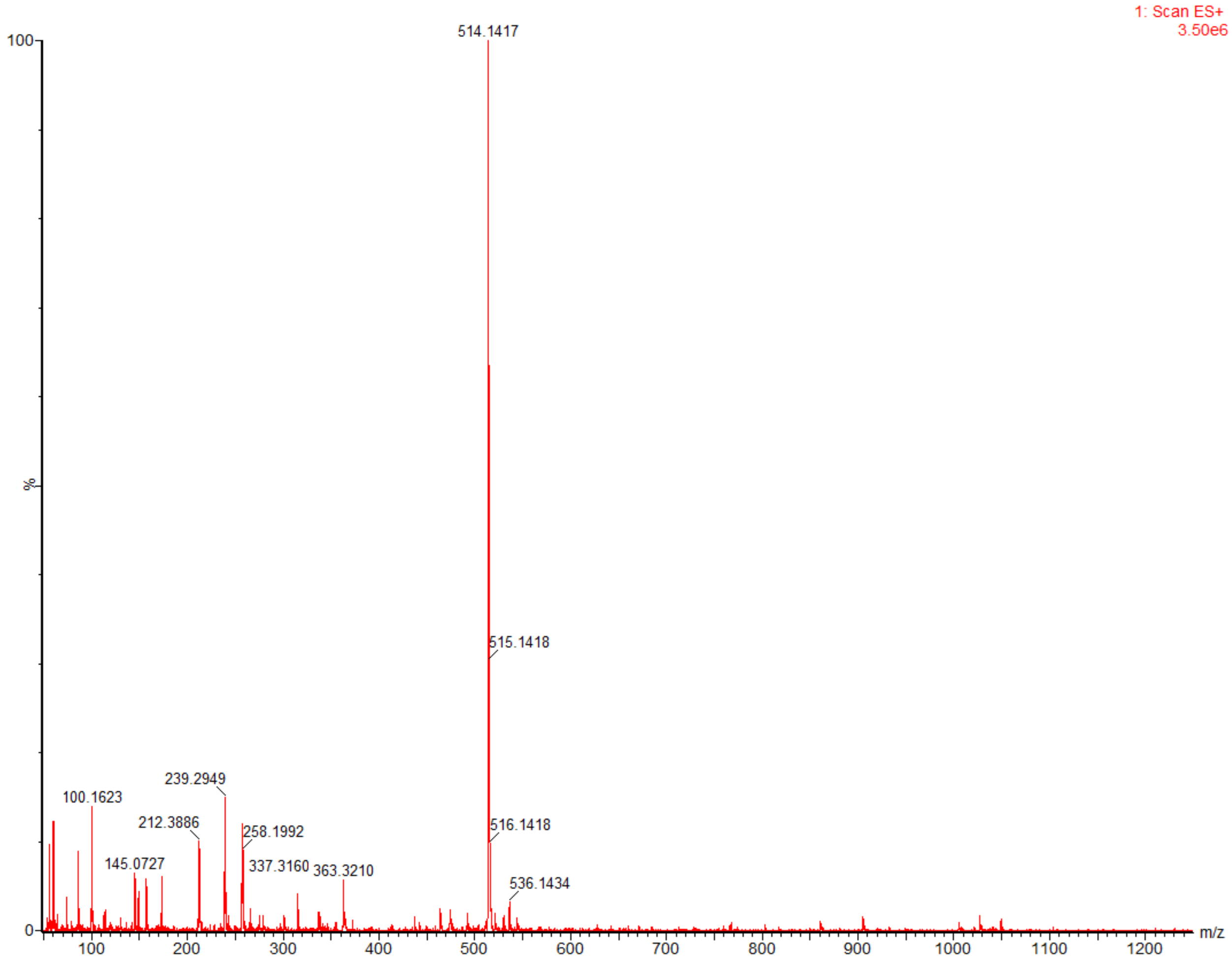
1: Scan ES+
3.50e6
514.1417
515.1418
516.1418
536.1434
100.1623
239.2949
212.3886
258.1992
145.0727
337.3160
363.3210
%
m/z

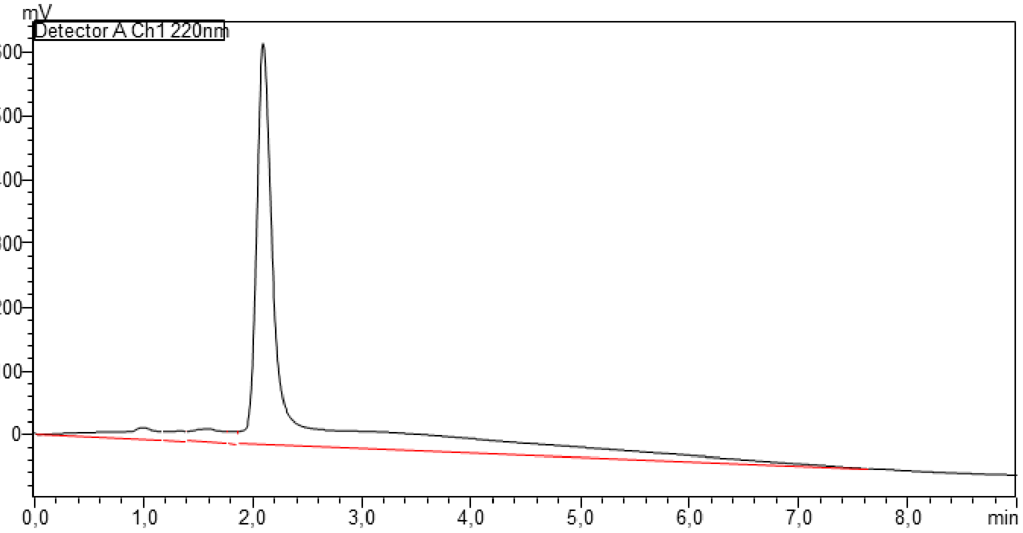
mV
Detector A Ch1 220nm
min

Figure S2. HR-ESI-MS of Peptide **4** ion [M+H]$^+$ and analytical HPLC trace at 220 nm.

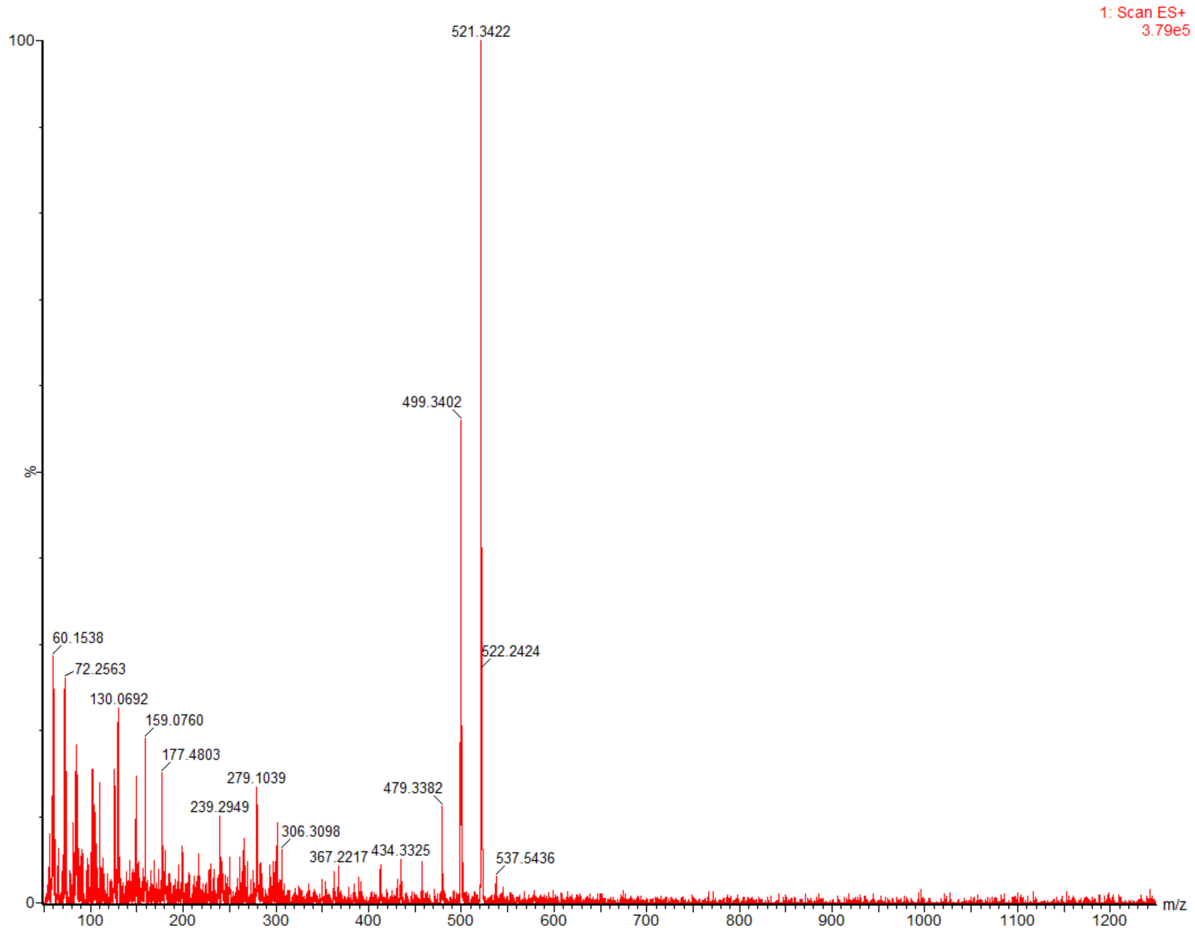

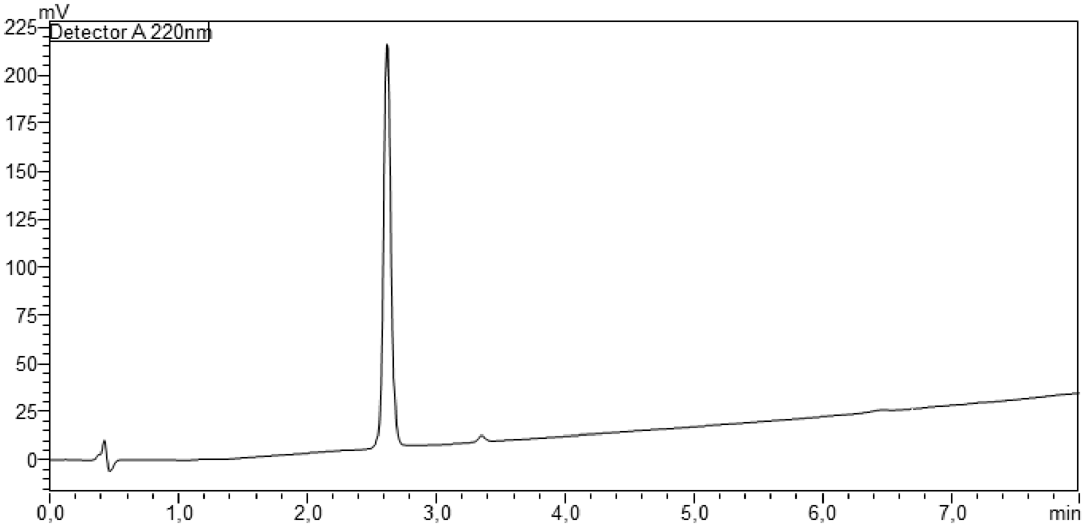

Figure S3. HR-ESI-MS of Peptide **5** ion $[M+H]^+$ and analytical HPLC trace at 220 nm.

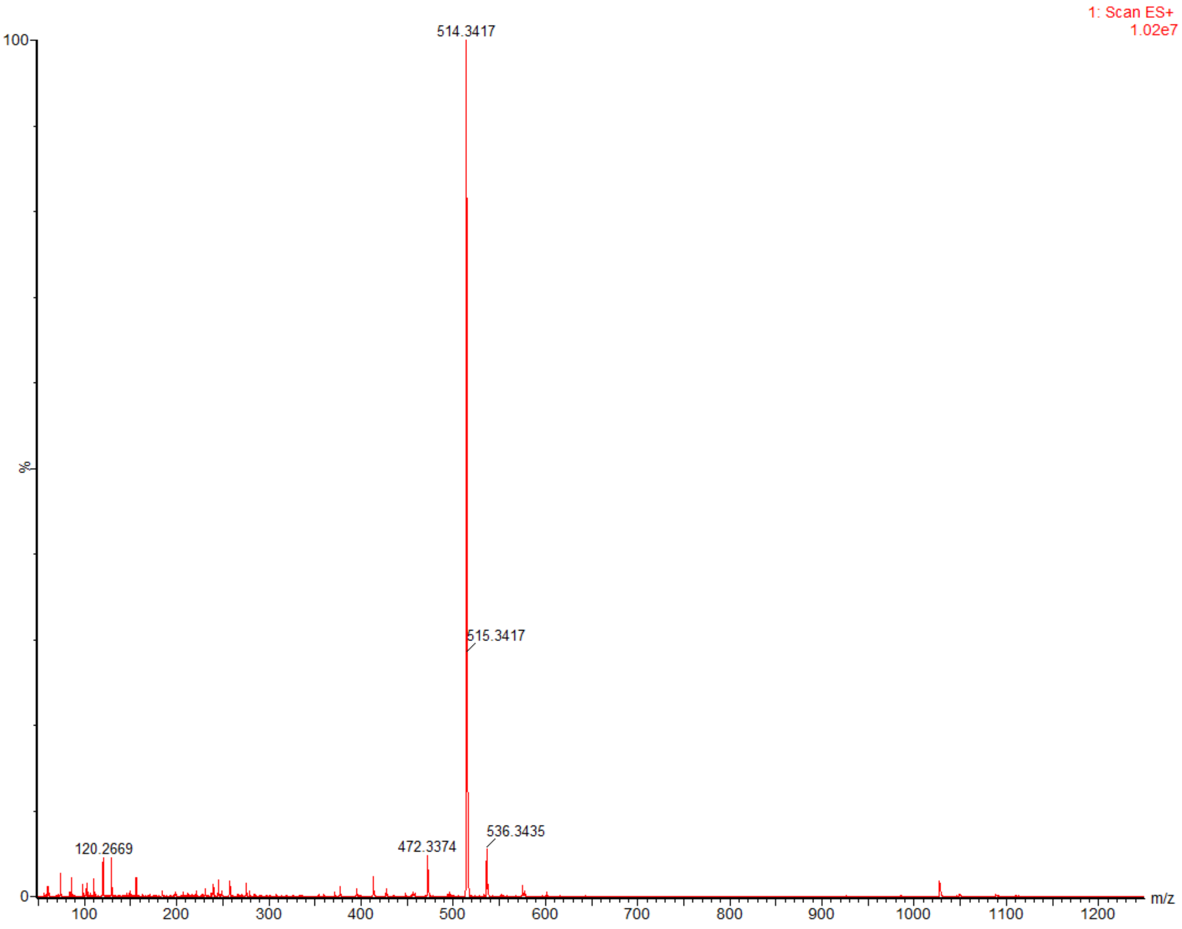

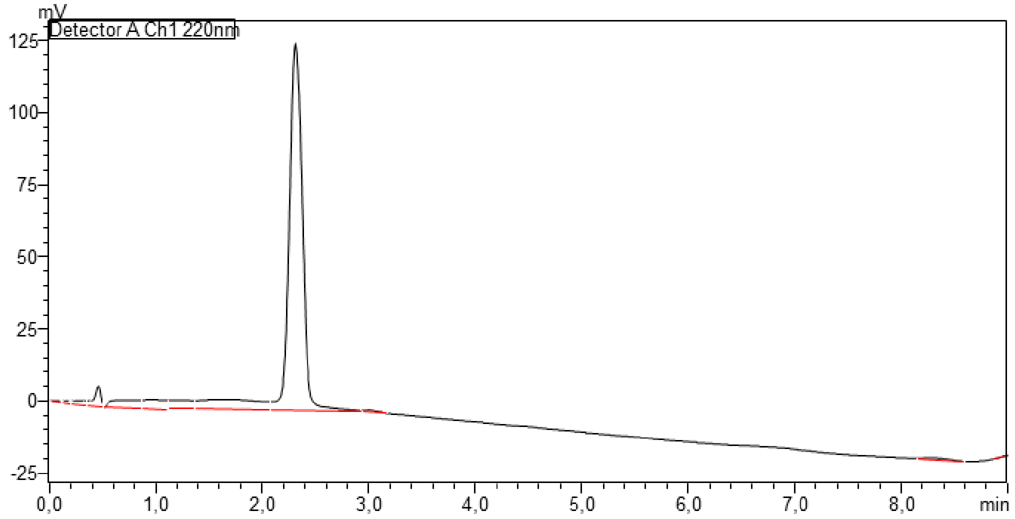

Figure S4. HR-ESI-MS of Peptide **6** ion $[M+H]^+$ and analytical HPLC trace at 220 nm.

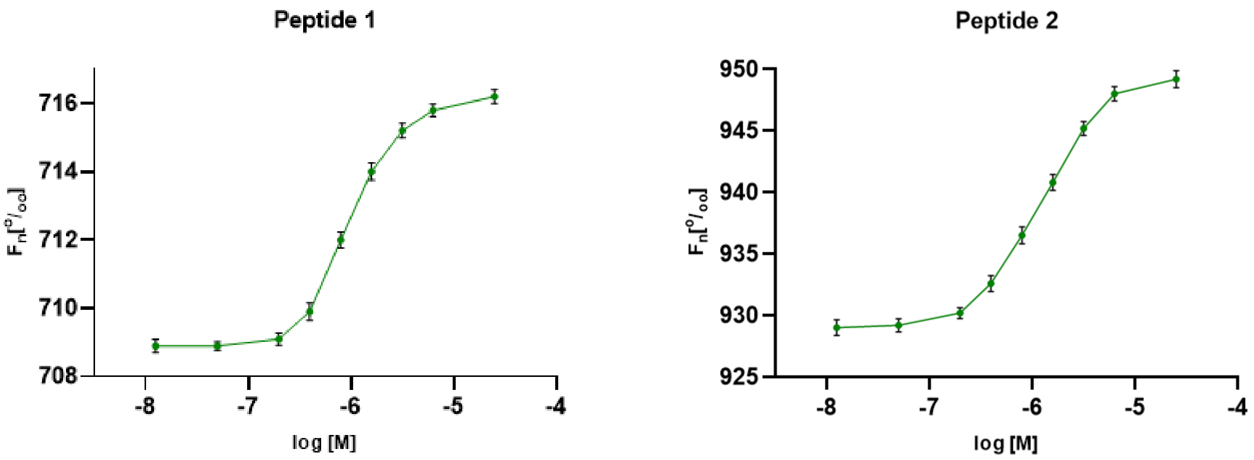

Figure S5. MST binding curve of peptide **1-2** to Spike 229E.

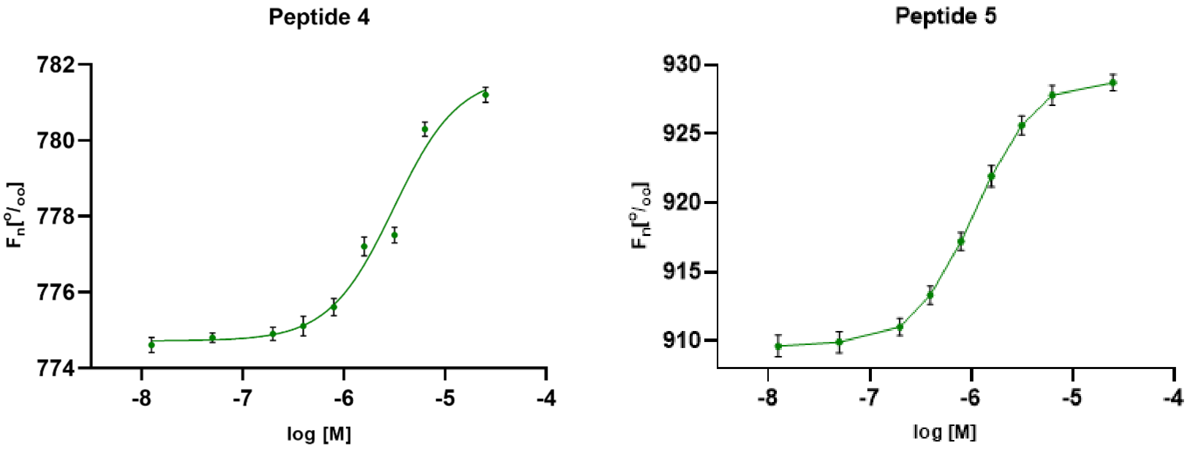

Figure S6. MST binding curve of peptide **4-5** to Spike 229E.

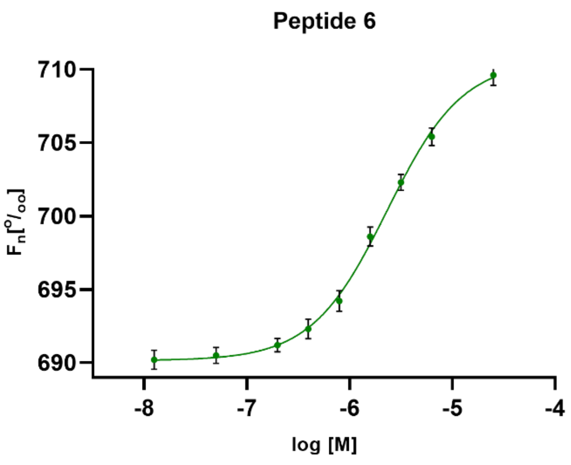

Figure S7. MST binding curve of peptide **6** to Spike 229E.

Table S2. NanoDSF assays were performed using the Prometheus. The peptides screening was performed at the fixed dose of 200 μM, with a protein concentration of 2 μM. For the analysis, standard capillaries were used.

Peptide	Start temp (°C)	End temp (°C)	Tm (°C)	Δ Tm (°C)
**HCoV-229E**	20	95	83.4	0
**1**	20	95	82.7	-0.7
**2**	20	95	82.5	-0.9

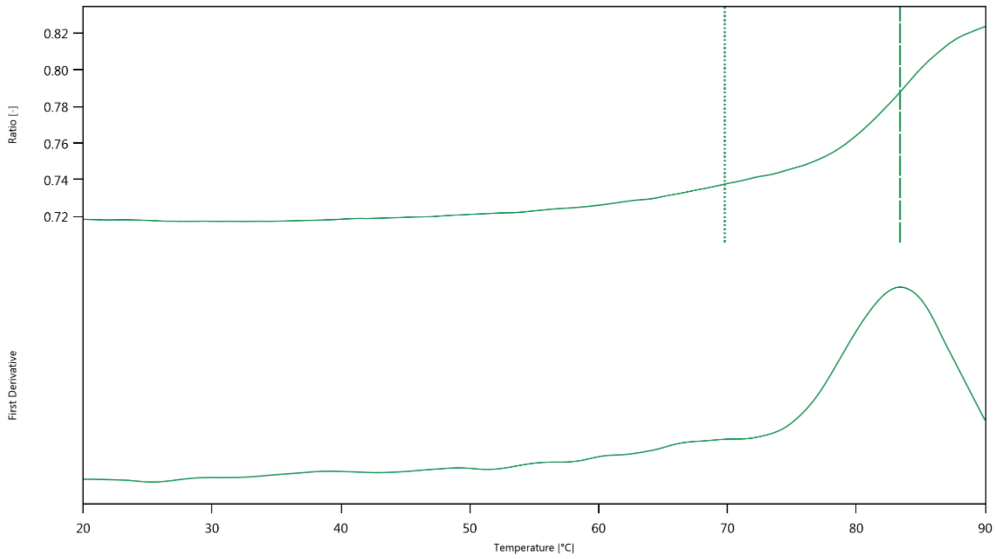

Figure S8. Melt curves and the first derivative of the melt curves of HCoV-229E. A ramp rate of 1.00 °C/min from 20°C through 90°C was used. Top: Fluorescence vs. temperature. Bottom: first derivative plot of the change in fluorescence vs. temperature. The median Boltzmann Tm and median derivative Tm values are shown as green dashed and green dotted vertical lines, respectively.

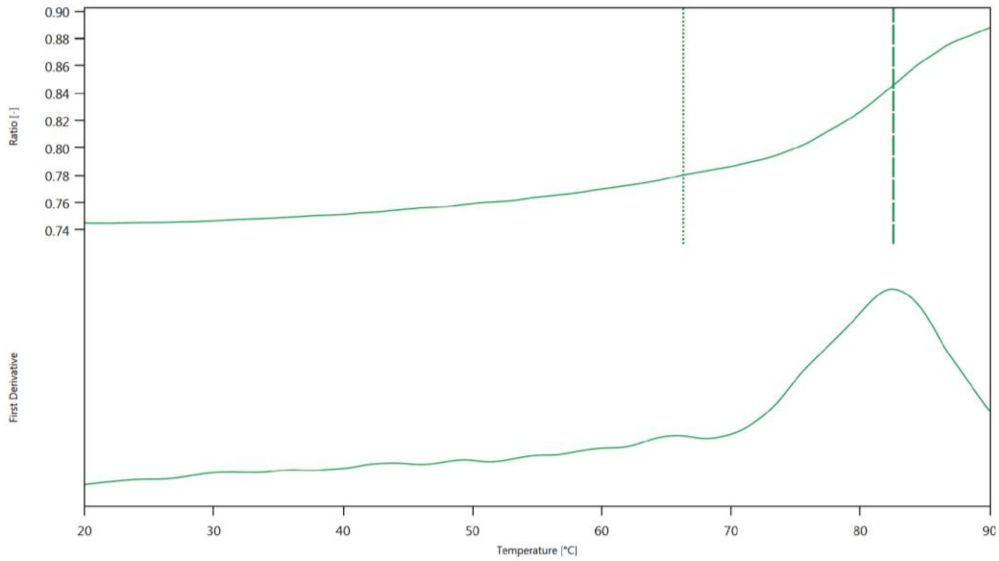

Figure S9. Melt curves and the first derivative of the melt curves of HCoV-229E-peptide **1**. A ramp rate of 1.00 °C/min from 20°C through 90°C was used. Top: Fluorescence vs. temperature. Bottom: first derivative plot of the change in fluorescence vs. temperature. The median Boltzmann Tm and median derivative Tm values are shown as green dashed and green dotted vertical lines, respectively.

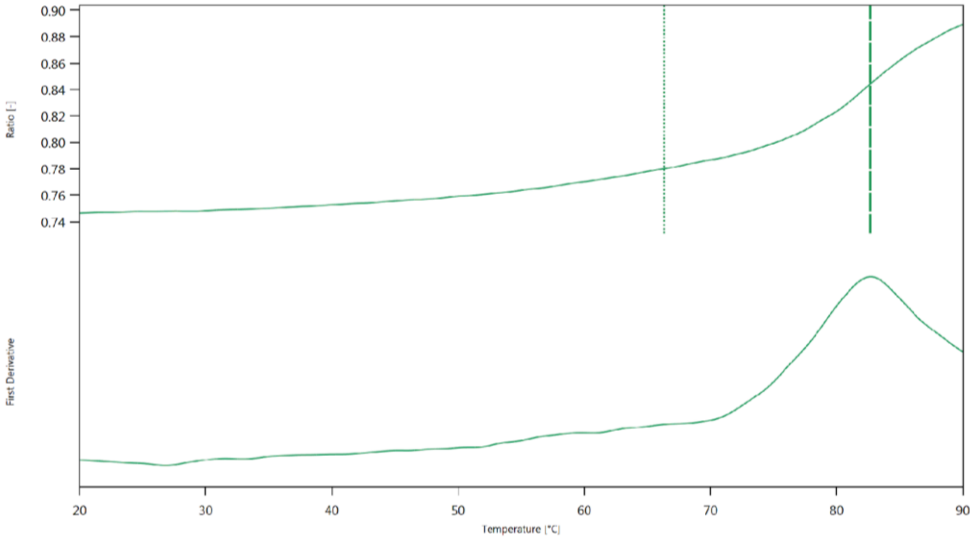

Figure S10. Melt curves and the first derivative of the melt curves of HCoV-229E-peptide **2**. A ramp rate of 1.00 °C/min from 20°C through 90°C was used. Top: Fluorescence vs. temperature. Bottom: first derivative plot of the change in fluorescence vs. temperature. The median Boltzmann Tm and median derivative Tm values are shown as green dashed and green dotted vertical lines, respectively.

Table S3. NanoDSF assays were performed using the Prometheus. The peptides screening was performed at the fixed dose of 200 μM, with a protein concentration of 2 μM. For the analysis, standard capillaries were used.

Peptide	Start temp (°C)	End temp (°C)	Tm (°C)	Δ Tm (°C)
**HCoV-229E**	20	95	81.6	0
**3**	20	95	81.9	0.3
**4**	20	95	81.7	0.1
**5**	20	95	82.5	0.9
**6**	20	95	82.0	0.4

Figure S8. Melt curves and the first derivative of the melt curves of HCoV-229E. A ramp rate of 1.00 °C/min from 20°C through 90°C was used. Top: Fluorescence vs. temperature. Bottom: first derivative plot of the change in fluorescence vs. temperature. The median Boltzmann Tm and median derivative Tm values are shown as green dashed and green dotted vertical lines, respectively.

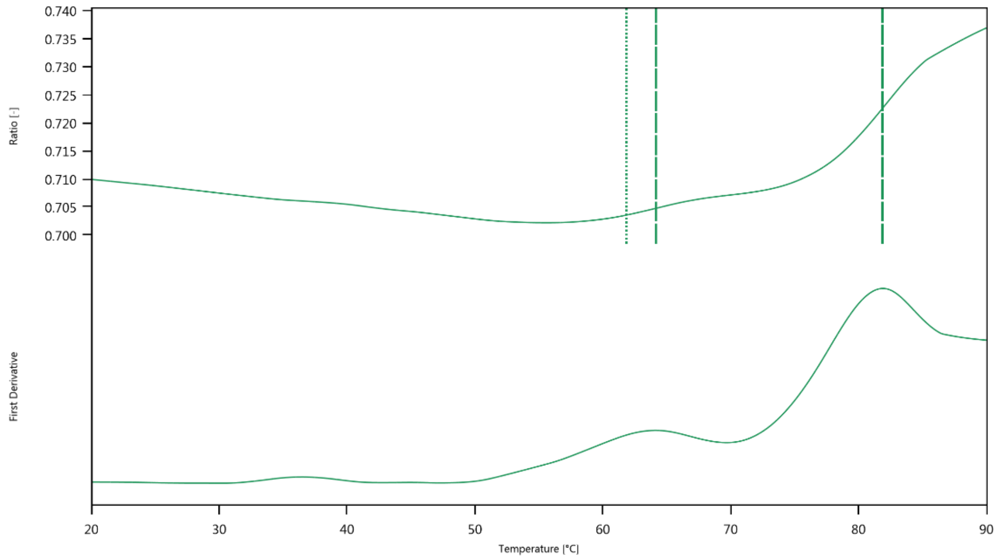

Figure S11. Melt curves and the first derivative of the melt curves of HCoV-229E-compound **3**. A ramp rate of 1.00 °C/min from 20°C through 90°C was used. Top: Fluorescence vs. temperature. Bottom: first derivative plot of the change in fluorescence vs. temperature. The median Boltzmann Tm and median derivative Tm values are shown as green dashed and green dotted vertical lines, respectively.

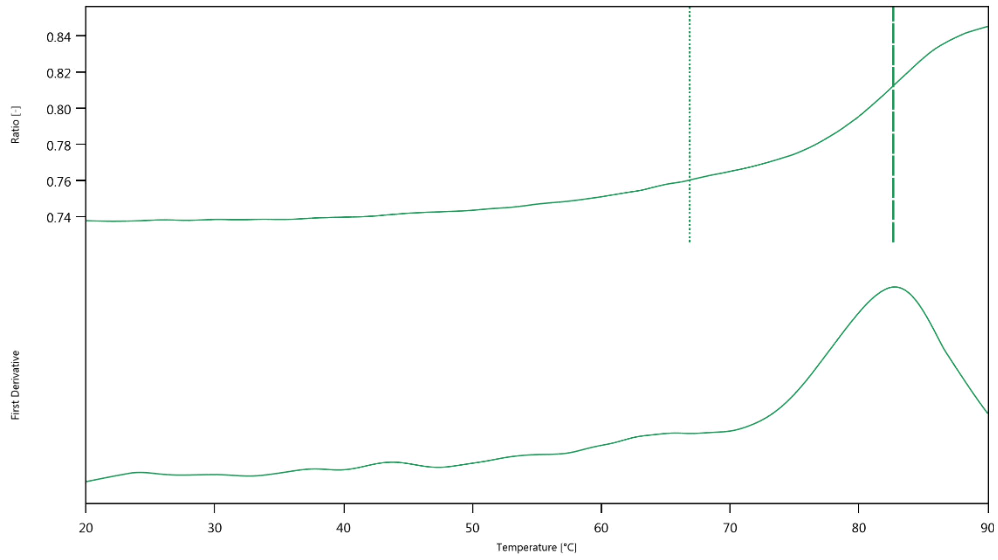

Figure S12. Melt curves and the first derivative of the melt curves of HCoV-229E-compound **4**. A ramp rate of 1.00 °C/min from 20°C through 90°C was used. Top: Fluorescence vs. temperature. Bottom: first derivative plot of the change in fluorescence vs. temperature. The median Boltzmann Tm and median derivative Tm values are shown as green dashed and green dotted vertical lines, respectively.

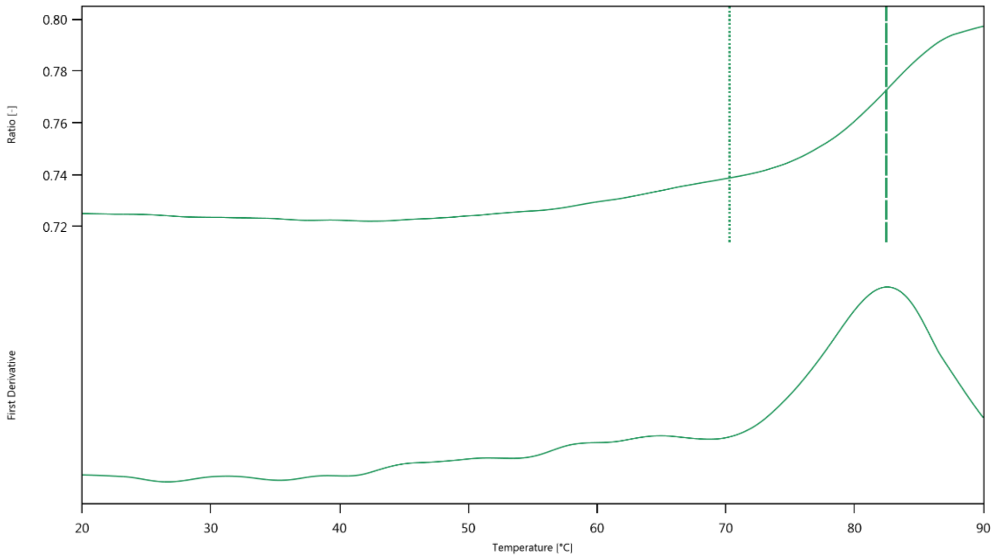

Figure S13. Melt curves and the first derivative of the melt curves of HCoV-229E-compound **5**. A ramp rate of 1.00 °C/min from 20°C through 90°C was used. Top: Fluorescence vs. temperature. Bottom: first derivative plot of the change in fluorescence vs. temperature. The median Boltzmann Tm and median derivative Tm values are shown as green dashed and green dotted vertical lines, respectively.

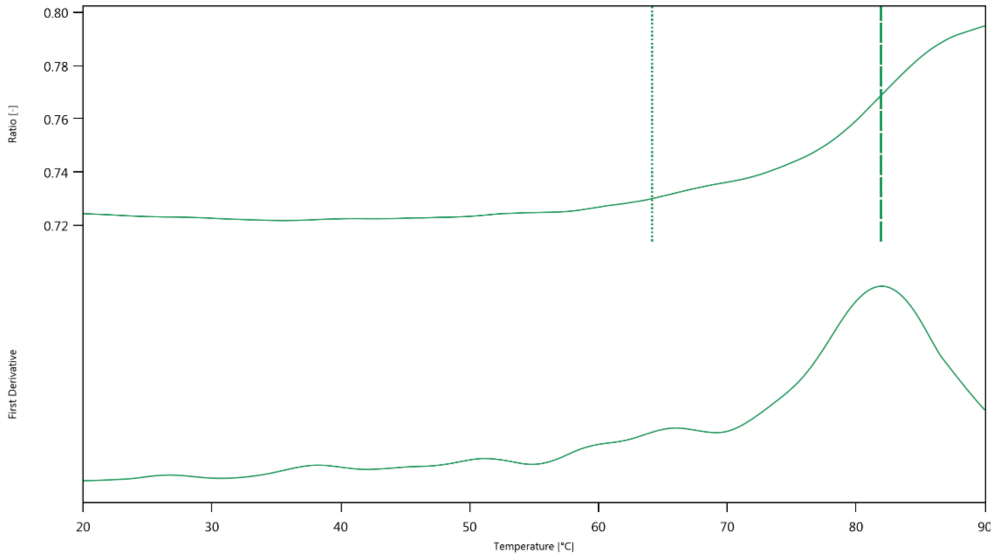

Figure S14. Melt curves and the first derivative of the melt curves of HCoV-229E-compound **6**. A ramp rate of 1.00 °C/min from 20°C through 90°C was used. Top: Fluorescence vs. temperature. Bottom: first derivative plot of the change in fluorescence vs. temperature. The median Boltzmann Tm and median derivative Tm values are shown as green dashed and green dotted vertical lines, respectively.

SiteMap study on the HCov229E spike protein.

The SiteMap analysis was carried out on the three available structures of the S protein as described in the experimental section. This mapping procedure identified the most promising sites for each structure.

Results were analyzed to find overlapping sites even if some difference is due to the diverse conformation represented by the structure.

The sites used for docking studies along with their score values are reported in Table S1. Figure S1 reports the structure of the spike protein with the representation of all 11 sites

Table S4. Identified and selected sites for the following docking studies on the three available structures. Calculated SiteMap scores are reported.

Title	Dscore	SiteScore	balance	contact	don/acc	enclosure	exposure	philic	phobic	volume
**7CYD_site_16**	0.938882	1.083224	0.306248	1.027663	0.941465	0.822441	0.410882	1.517126	0.464617	661.175375
**7CYD_ site_5**	1.152472	1.101032	2.482874	1.070801	1.169560	0.769494	0.651270	0.764491	1.898135	716.784250
**7CYC _site_7**	1.129490	1.147268	1.939785	1.145289	0.597249	0.918204	0.497525	1.108689	2.150618	664.176625
**7CYD_site_19**	1.129047	1.108210	1.826333	1.120222	0.885810	0.835996	0.398148	0.958746	1.750989	516.215000
**6u7h_ site_6**	1.091245	1.121868	1.714181	1.211707	0.786582	0.880225	0.585657	1.156105	1.981773	469.181125
**6u7h_site_3**	1.062063	1.127712	0.944705	1.167400	0.696023	0.888963	0.501466	1.261702	1.191936	591.760750
**7CYC _site_8**	1.048325	1.120879	0.672164	1.115720	0.884789	0.878746	0.437673	1.285039	0.863757	651.099750
**6u7h_site_18**	1.048191	1.075254	0.681866	1.067119	1.053646	0.810524	0.461818	1.159887	0.790888	543.783625
**6u7h_site_10**	1.046926	1.012879	0.848613	0.911779	1.046591	0.687085	0.601460	0.936580	0.794794	758.887500
**7CYC_site_20**	1.038268	0.996920	0.626616	0.803172	0.784355	0.652655	0.638835	0.899772	0.563812	597.849000
**6u7h_site_17**	1.006332	1.130177	0.590543	1.241054	1.073772	0.892650	0.425856	1.439442	0.850052	377.042750

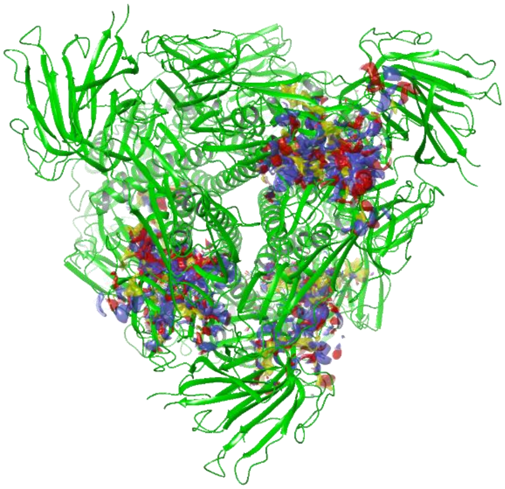

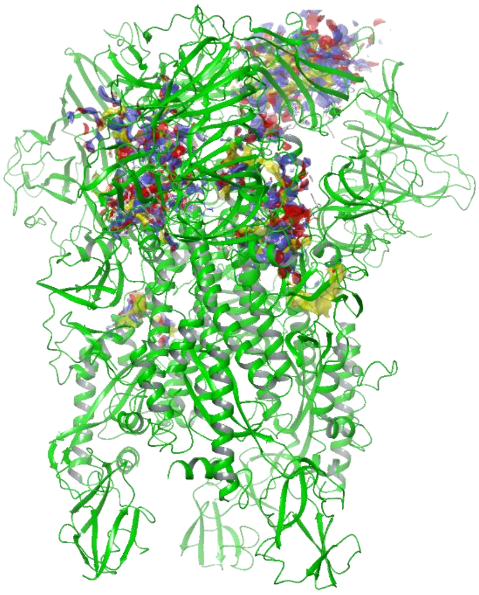

Figure S15. Top view (left) and side view (right) of the S protein (green cartoon). The position of all identified and studies sited are reported. Sites are depicted with their isosurfaces for the dry (yellow), the H-bond donor (blu), and the H-bond acceptor (red) probes.

Table S5. Docking score values obtained by compounds **5** in the eleven sites of the S protein.

ID	Sequence	6UH7 site 3	6UH7 site 6	6UH7 site 10	6UH7 site 17	6UH7 site 18	7CYC site 7	7CYC site 8	7CYC site 20	7CYD site 5	7CYD site 16	7CYD site 19
**5**	S*N*KHS	-8.522	-6.742	-6.892	-8.089	-7.104	-6.039	**-9.796**	-7.246	-6.935	-6.544	-4.985
